# Significance of body temperature in elderly patients with sepsis

**DOI:** 10.1186/s13054-020-02976-6

**Published:** 2020-06-30

**Authors:** Takashi Shimazui, Taka-aki Nakada, Keith R. Walley, Taku Oshima, Toshikazu Abe, Hiroshi Ogura, Atsushi Shiraishi, Shigeki Kushimoto, Daizoh Saitoh, Seitaro Fujishima, Toshihiko Mayumi, Yasukazu Shiino, Takehiko Tarui, Toru Hifumi, Yasuhiro Otomo, Kohji Okamoto, Yutaka Umemura, Joji Kotani, Yuichiro Sakamoto, Junichi Sasaki, Shin-ichiro Shiraishi, Kiyotsugu Takuma, Ryosuke Tsuruta, Akiyoshi Hagiwara, Kazuma Yamakawa, Tomohiko Masuno, Naoshi Takeyama, Norio Yamashita, Hiroto Ikeda, Masashi Ueyama, Satoshi Fujimi, Satoshi Gando, on behalf of the JAAM FORECAST Group, Takashi Shimazui, Taka-aki Nakada, Keith R. Walley, Taku Oshima, Toshikazu Abe, Hiroshi Ogura, Atsushi Shiraishi, Shigeki Kushimoto, Daizoh Saitoh, Seitaro Fujishima, Toshihiko Mayumi, Yasukazu Shiino, Takehiko Tarui, Toru Hifumi, Yasuhiro Otomo, Kohji Okamoto, Yutaka Umemura, Joji Kotani, Yuichiro Sakamoto, Junichi Sasaki, Shin-ichiro Shiraishi, Kiyotsugu Takuma, Ryosuke Tsuruta, Akiyoshi Hagiwara, Kazuma Yamakawa, Tomohiko Masuno, Naoshi Takeyama, Norio Yamashita, Hiroto Ikeda, Masashi Ueyama, Satoshi Fujimi, Satoshi Gando, Osamu Tasaki, Yasumitsu Mizobata, Hiraku Funakoshi, Toshiro Okuyama, Iwao Yamashita, Toshio Kanai, Yasuo Yamada, Mayuki Aibiki, Keiji Sato, Susumu Yamashita, Kenichi Yoshida, Shunji Kasaoka, Akihide Kon, Hiroshi Rinka, Hiroshi Kato, Hiroshi Okudera, Eichi Narimatsu, Toshifumi Fujiwara, Manabu Sugita, Yasuo Shichinohe, Hajime Nakae, Ryouji Iiduka, Mitsunobu Nakamura, Yuji Murata, Yoshitake Sato, Hiroyasu Ishikura, Yasuhiro Myojo, Yasuyuki Tsujita, Kosaku Kinoshita, Hiroyuki Yamaguchi, Toshihiro Sakurai, Satoru Miyatake, Takao Saotome, Susumu Yasuda, Toshikazu Abe, Hiroshi Ogura, Yutaka Umemura, Atsushi Shiraishi, Shigeki Kushimoto, Daizoh Saitoh, Seitaro Fujishima, Junichi Sasaki, Toshihiko Mayumi, Yasukazu Shiino, Taka-aki Nakada, Takehiko Tarui, Toru Hifumi, Yasuhiro Otomo, Joji Kotani, Yuichiro Sakamoto, Shin-ichiro Shiraishi, Kiyotsugu Takuma, Ryosuke Tsuruta, Akiyoshi Hagiwara, Kazuma Yamakawa, Naoshi Takeyama, Norio Yamashita, Hiroto Ikeda, Yasuaki Mizushima, Satoshi Gando

**Affiliations:** 1 Department of Emergency and Critical Care Medicine, Chiba University Graduate School of Medicine, 1-8-1 Inohana, Chuo, Chiba 260-8677 Japan; 2 Centre for Heart Lung Innovation, University of British Columbia, Vancouver, Canada; 3 Department of General Medicine, Juntendo University, Tokyo, Japan; 4 Health Services Research and Development Center, University of Tsukuba, Tsukuba, Japan; 5 Department of Traumatology and Acute Critical Medicine, Osaka University Graduate School of Medicine, Osaka, Japan; 6 Emergency and Trauma Center, Kameda Medical Center, Kamogawa, Japan; 7 Division of Emergency and Critical Care Medicine, Tohoku University Graduate School of Medicine, Sendai, Japan; 8 Division of Traumatology, Research Institute, National Defense Medical College, Tokorozawa, Japan; 9 Center for General Medicine Education, Keio University School of Medicine, Tokyo, Japan; 10 Department of Emergency Medicine, School of Medicine, University of Occupational and Environmental Health, Kitakyushu, Japan; 11 Department of Acute Medicine, Kawasaki Medical School, Kurashiki, Japan; 12 Department of Trauma and Critical Care Medicine, Kyorin University School of Medicine, Mitaka, Japan; 13 Department of Emergency and Critical Care Medicine, St. Luke’s International Hospital, Tokyo, Japan; 14 Trauma and Acute Critical Care Center, Medical Hospital, Tokyo Medical and Dental University, Tokyo, Japan; 15 Department of Surgery, Center for Gastroenterology and Liver Disease, Kitakyushu City Yahata Hospital, Kitakyushu, Japan; 16 Division of Disaster and Emergency Medicine, Department of Surgery Related, Kobe University Graduate School of Medicine, Kobe, Japan; 17 Emergency and Critical Care Medicine, Saga University Hospital, Saga, Japan; 18 Department of Emergency and Critical Care Medicine, Keio University School of Medicine, Tokyo, Japan; 19 Department of Emergency and Critical Care Medicine, Aizu Chuo Hospital, Aizuwakamatsu, Japan; 20 Emergency & Critical Care Center, Kawasaki Municipal Kawasaki Hospital, Kawasaki, Japan; 21 Advanced Medical Emergency & Critical Care Center, Yamaguchi University Hospital, Ube, Japan; 22 Center Hospital of the National Center for Global Health and Medicine, Tokyo, Japan; 23 Division of Trauma and Surgical Critical Care, Osaka General Medical Center, Osaka, Japan; 24 Department of Emergency and Critical Care Medicine, Nippon Medical School, Tokyo, Japan; 25 Advanced Critical Care Center, Aichi Medical University Hospital, Nagakute, Japan; 26 Advanced Emergency Medical Service Center, Kurume University Hospital, Kurume, Japan; 27 Department of Emergency Medicine, Teikyo University School of Medicine, Tokyo, Japan; 28 Department of Trauma, Critical Care Medicine, and Burn Center, Japan Community Healthcare Organization, Chukyo Hospital, Nagoya, Japan; 29 Division of Acute and Critical Care Medicine, Hokkaido University Graduate School of Medicine, Sapporo, Japan; 30 Acute and Critical Care Center, Department of Acute and Critical Care Medicine, Sapporo Higashi Tokushukai Hospital, Sapporo, Japan

**Keywords:** Septic shock, Elderly, Body temperature, Fever, Hypothermia

## Abstract

**Background:**

Elderly patients have a blunted host response, which may influence vital signs and clinical outcomes of sepsis. This study was aimed to investigate whether the associations between the vital signs and mortality are different in elderly and non-elderly patients with sepsis.

**Methods:**

This was a retrospective observational study. A Japanese multicenter sepsis cohort (FORECAST, *n* = 1148) was used for the discovery analyses. Significant discovery results were tested for replication using two validation cohorts of sepsis (JAAMSR, Japan, *n* = 624; SPH, Canada, *n* = 1004). Patients were categorized into elderly and non-elderly groups (age ≥ 75 or < 75 years). We tested for association between vital signs (body temperature [BT], heart rate, mean arterial pressure, systolic blood pressure, and respiratory rate) and 90-day in-hospital mortality (primary outcome).

**Results:**

In the discovery cohort, non-elderly patients with BT < 36.0 °C had significantly increased 90-day mortality (*P* = 0.025, adjusted hazard ratio 1.70, 95% CI 1.07–2.71). In the validation cohorts, non-elderly patients with BT < 36.0 °C had significantly increased mortality (JAAMSR, *P* = 0.0024, adjusted hazard ratio 2.05, 95% CI 1.29–3.26; SPH, *P* = 0.029, adjusted hazard ratio 1.36, 95% CI 1.03–1.80). These differences were not observed in elderly patients in the three cohorts. Associations between the other four vital signs and mortality were not different in elderly and non-elderly patients. The interaction of age and hypothermia/fever was significant (*P* < 0.05).

**Conclusions:**

In septic patients, we found mortality in non-elderly sepsis patients was increased with hypothermia and decreased with fever. However, mortality in elderly patients was not associated with BT. These results illuminate the difference in the inflammatory response of the elderly compared to non-elderly sepsis patients.

## Background

A dysregulated host response to infection is a cornerstone of the pathophysiology of sepsis [1, 2]. A dysregulated response alters vital signs such as body temperature (BT), heart rate (HR), blood pressure (BP), and respiratory rate (RR) [3, 4]. Alterations such as hypothermia, fever, tachycardia, hypotension, or tachypnea tend to be associated with altered clinical outcomes [5–9].

Elderly patients generally have a blunted host inflammatory response [10–12], which could contribute to different associations between the vital signs and mortality between non-elderly and elderly patients, as observed in a variety of pathological settings [13–15]. In sepsis, a single-center study with a limited sample size demonstrated that BT, as a component of the vital signs, is an independent predictor of mortality only in elderly patients [13]. However, whether the relationships between altered vital signs and clinical outcomes differ between elderly and non-elderly patients remains unclear. Investigating and clarifying these differences might contribute to improving the quality of sepsis care, thus reducing mortality rates in critically ill septic patients.

Thus, we hypothesized that associations between initial vital signs and mortality are different for elderly and non-elderly patients with sepsis. Three large sepsis cohorts were investigated to assess the primary outcome of 90-day in-hospital mortality.

## Materials and methods

### Study cohorts

The current observational study was retrospectively conducted using three sepsis cohorts. Written informed consent was waived because of the study design.

### Discovery cohort: FORECAST cohort

FORECAST (Focused Outcomes Research in Emergency Care in Acute Respiratory Distress Syndrome, Sepsis, and Trauma) was a multicenter (ICUs, *n* = 59, Japan), prospective observational study conducted by the Japanese Association for Acute Medicine (JAAM) from January 2016 to March 2017 [16]. Of the 1184 patients with sepsis, 1148 patients who had records of 90-day in-hospital mortality were included in the analyses. The Institutional Review Board of Chiba University Graduate School of Medicine has approved the study (approval number, 3407).

### Validation cohort 1: JAAMSR cohort

JAAMSR (Japanese Association for Acute Medicine Sepsis Registry) was a multicenter (ICUs, *n* = 15, Japan), prospective observational study conducted by the JAAM from June 2010 to May 2011 [17]. Some of the clinical centers were included in both JAAMSR and FORECAST cohorts; however, not all were included in both of these cohorts. All 624 patients screened with sepsis were included in the analyses. The Institutional Review Board of Chiba University Graduate School of Medicine has approved the study (approval number, 3407).

### Validation cohort 2: SPH cohort

Patients admitted to the ICU at St. Paul’s Hospital (SPH) in Vancouver, Canada, between July 2000 and January 2004 (*n* = 1337) were screened [18]. Of these, 1037 patients met the definition of sepsis on ICU admission. Of these, 1004 patients had records of 90-day in-hospital mortality and were included in the analyses. The Institutional Review Board of SPH has approved the study (approval number, H02-50076).

### Data collection and definitions

Sepsis and septic shock were defined based on the sepsis-2 criteria [19]. According to the current standard [1], severe sepsis according to the sepsis-2 criteria was assumed as sepsis. Patients with age ≥ 75 years were defined as elderly and < 75 years as non-elderly, based on previous reports [20–22]. In this study, the vital signs included BT, HR, mean arterial pressure (MAP), systolic blood pressure (SBP), and RR. The worst vital sign values corresponding to the APACHE II scoring were retrieved within 24 h of the diagnosis of sepsis in all cohorts. Each vital sign was divided into two groups based on the cutoff values from the sepsis-2 and sepsis-3 criteria [1, 19] (additional details are provided in Additional file 1: Table S1). The definition of the suspected site of infection was applied as described elsewhere [16].

### Statistical analysis

Our primary analysis used Cox regression to test for hazard of death over 90 days by the vital signs in the elderly and non-elderly groups. We selected this approach to adjust for potential baseline imbalances, including age (per year), sex, chronic steroid use, and APACHE II score (Additional file 1: Table S2, correlation analysis) based on previous reports [18, 23]. Significant discovery results were tested for replication and generalizability in patients of the same ancestry in validation cohort 1 and patients of different ancestry in validation cohort 2. We tested for homogeneity of results across cohorts using a meta-analytic technique. Sensitivity analysis using the Cox regression was conducted by adding parameters with significant difference between the non-elderly and elderly patients including comorbidities as covariates. Sensitivity analyses using age cutoffs of 65, 70, and 80 years old for significant discovery results were conducted [24–28]. Interactions between age and BT for mortality were tested using Cox regression analysis. In addition, the Cox regression analysis for 28-day in-hospital mortality was conducted.

Univariate analysis was performed using Pearson’s chi-square test or a Mann-Whitney *U* test. Two-tailed *P* values < 0.05 were considered to be significant. Analyses were performed using either the SPSS software version 24.0 (IBM Corporation, Armonk, NY, USA) or the Review Manager (RevMan) version 5.3 software (The Nordic Cochrane Centre, The Cochrane Collaboration, Copenhagen, Denmark).

## Results

There were 628 non-elderly and 520 elderly patients in the discovery FORECAST cohort (Table 1 and Additional file 1: Table S3). Of the vital sign variables on day 1, elderly patients had significantly lower BT and HR compared to non-elderly patients. Elderly patients also had significantly higher 90-day mortality compared to non-elderly patients (Table 1).

**Table 1:** Baseline characteristics and clinical outcomes in the derivation cohort (FORECAST cohort)

	Non-elderly (< 75 years) (*n* = 628)	Elderly (≥ 75 years) (*n* = 520)	*P* value
Characteristics			
Age, years	65 (55–70)	82 (78–86)	< 0.0001
Male sex, *n* (%)	405 (64.5)	288 (55.4)	0.0017
Suspected site of infection, *n* (%)			
Lung	193 (30.7)	164 (31.5)	0.77
Intra-abdominal	150 (23.9)	145 (27.9)	0.12
Urinary tract	102 (16.2)	116 (22.3)	0.0091
Soft tissue	79 (12.6)	35 (6.7)	0.0010
Others^a^	104 (16.6)	60 (11.5)	0.015
Septic shock, *n* (%)	387 (61.6)	331 (63.7)	0.48
Body mass index	22.5 (19.3–25.2)	20.8 (18.6–23.9)	< 0.0001
Chronic steroid use, *n* (%)	86 (13.7)	55 (10.6)	0.11
Comorbidity, *n* (%)			
Diabetes mellitus	155 (24.7)	107 (20.6)	0.099
Stroke	55 (8.8)	78 (15.0)	0.0010
Malignancy	101 (16.1)	77 (14.8)	0.55
Heart failure	49 (7.8)	74 (14.2)	0.0005
Chronic kidney disease	39 (6.2)	41 (7.9)	0.27
Liver disease	43 (6.8)	25 (4.8)	0.15
Chronic lung disease	39 (6.2)	41 (7.9)	0.27
Charlson comorbidity index	1 (0–2)	1 (0–2)	0.014
SOFA score	9 (6–11)	9 (6–11)	0.73
APACHE II score	22 (16–29)	23 (18–30)	0.085
Vital signs on day 1^b^			
Body temperature, °C	38.0 (36.8–39.0)	37.6 (36.7–38.5)	0.0003
Heart rate, beats/min	114 (96–133)	108 (91–123)	< 0.0001
Mean arterial pressure, mmHg	63 (53–78)	63 (52–78)	0.65
Systolic blood pressure, mmHg	86 (72–110)	90 (72–110)	0.38
Respiratory rate, breath/min	25 (20–32)	25 (20–32)	0.63
Outcome			
28-day in-hospital mortality, *n* (%)	90 (14.3)	100 (19.2)	0.026
90-day in-hospital mortality, *n* (%)	123 (19.6)	130 (25.0)	0.028

Median (interquartile range)

*SOFA* sequential organ failure assessment, *APACHE* acute physiology and chronic health evaluation

^a^Including central nervous system, catheter-related, osteoarticular, endocardium, wound, implant device-related, and undifferentiated infection

^b^Most abnormal value corresponding to the APACHE II score

*P* values were calculated using Pearson’s chi-square test and Mann-Whitney *U* test

### Discovery analysis for all vital signs (FORECAST cohort)

**Fig. 1:**
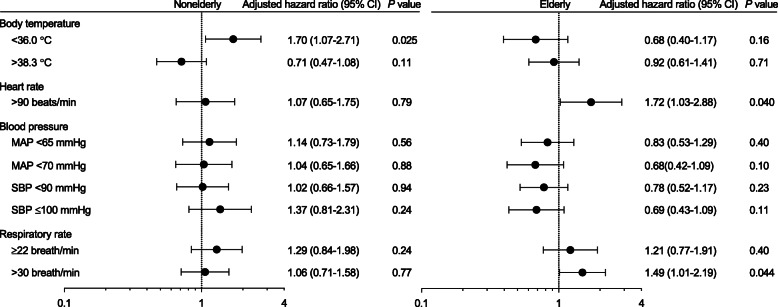
Associations between the vital signs and 90-day in-hospital mortality in discovery cohort (FORECAST cohort). Non-elderly patients with BT < 36.0 °C showed a significant increase for hazard of death over 90-day period. The hypothermia was not associated with mortality in elderly patients. Heart rate > 90 beats/min and RR > 30 breaths/min had a significant association for increased mortality in elderly patients. CI, confidence interval. The adjusted hazard ratio was calculated using potentially confounding factors such as the age, sex, chronic steroid use, and acute physiology and chronic health evaluation (APACHE) II score

**Fig. 2:**
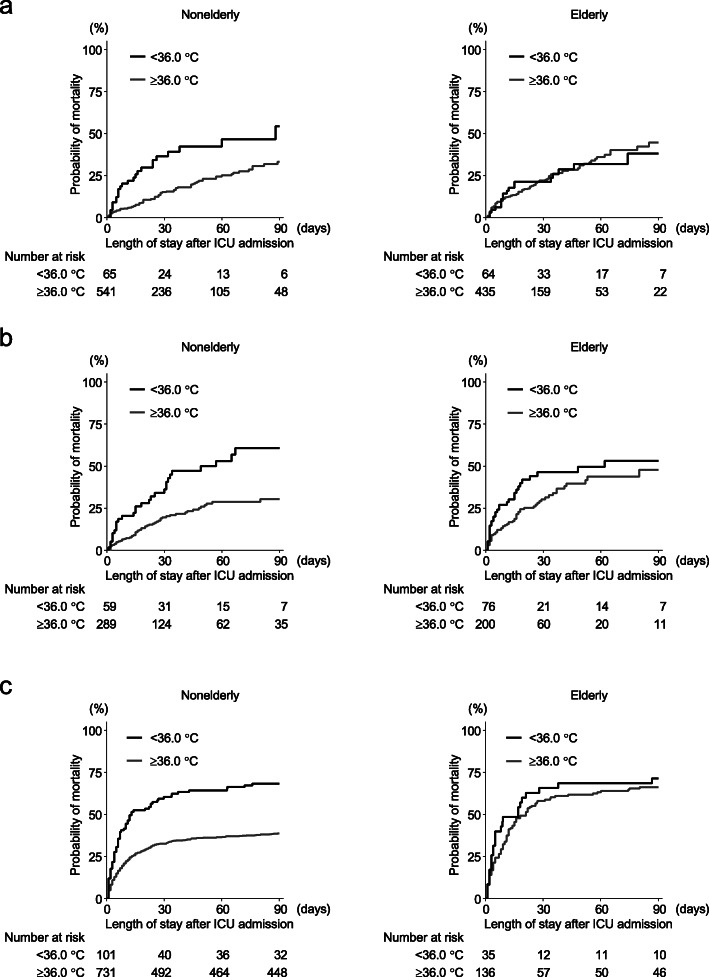
Probability of mortality corresponding to the body temperature category of < 36.0 °C. **a** Discovery cohort (FORECAST cohort). **b** Validation cohort 1 (JAAMSR cohort). **c** Validation cohort 2 (SPH cohort). Non-elderly patients with hypothermia had higher mortality in all three cohorts

In the primary analysis of the five vital signs using Cox regression, non-elderly patients with BT < 36.0 °C had a significantly increased hazard of death over 90 days (*P* = 0.025, adjusted hazard ratio 1.70, 95% CI 1.07–2.71) (Fig. 1, Fig. 2a). In contrast, elderly patients with hypothermia did not show differences in 90-day mortality (*P* = 0.16) (Fig. 1, Fig. 2a). In elderly patients, HR > 90 beats/min and RR > 30 breaths/min were associated with increased mortality (HR, *P* = 0.040, adjusted hazard ratio 1.72, 95% CI 1.03–2.88; RR, *P* = 0.044, adjusted hazard ratio 1.49, 95% CI 1.01–2.19) (Fig. 1).

### Validation of significant discovery results (JAAMSR, SPH cohorts)

**Fig. 3:**
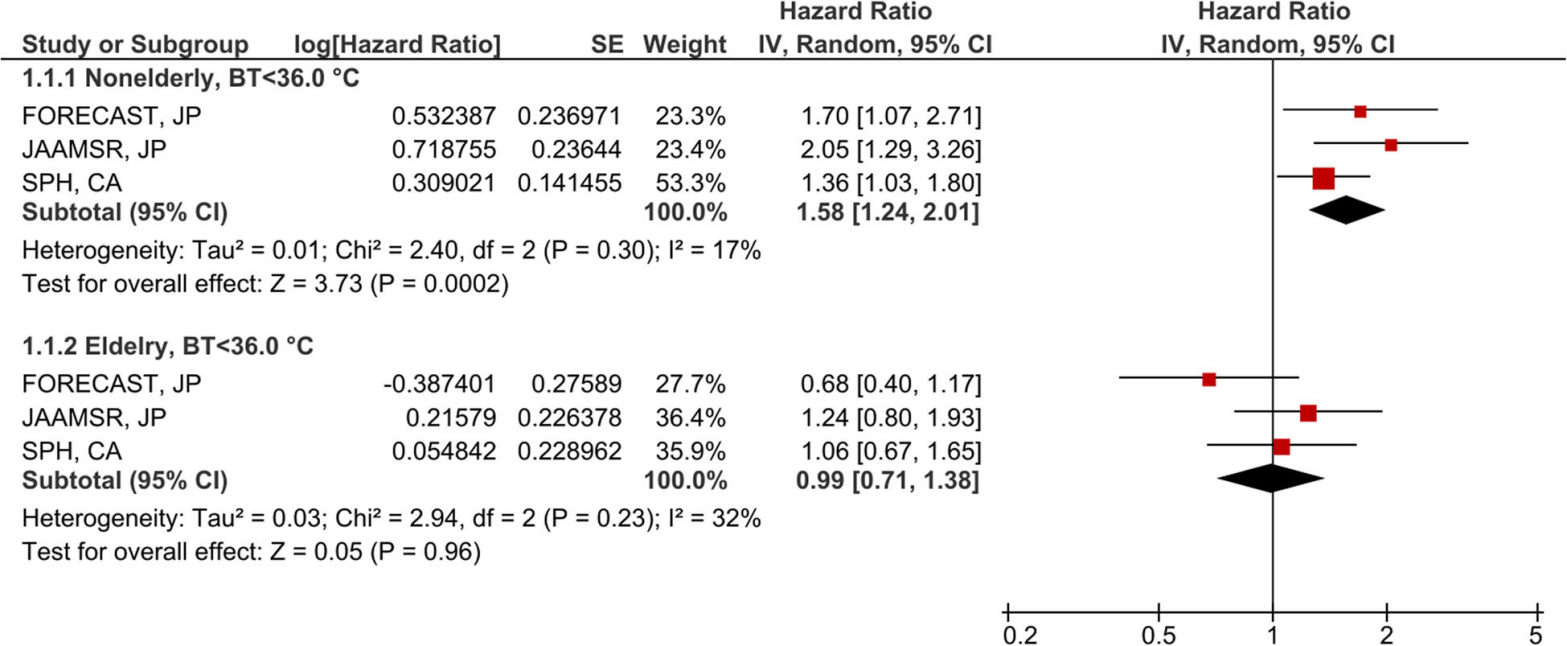
Meta-analysis to test for the homogeneity between each study for association of body temperature < 36.0 °C and 90-day in-hospital mortality. Non-elderly patients revealed no significant differences in effect between all three cohorts (*I*^2^ = 17%) and a significant combined effect (*P* = 0.0002, hazard ratio 1.58, 95% CI 1.24–2.01). JP, Japan; CA, Canada; CI, confidence interval. The hazard ratio from each cohorts was calculated with adjusting the potentially confounding factors such as the age, sex, chronic steroid use, and acute physiology and chronic health evaluation (APACHE) II score

Non-elderly patients with BT < 36.0 °C showed significantly increased mortality in validation cohort 1 (Japan) (JAAMSR, *P* = 0.0024, adjusted hazard ratio 2.05, 95% CI 1.29–3.26) (Fig. 2b) (Additional file 1: Table S4, Baseline characteristics). The same effect was replicated in the patients of different ancestry in validation cohort 2 (Canada) (SPH, *P* = 0.029, adjusted hazard ratio 1.36, 95% CI 1.03–1.80) (Fig. 2c) (Additional file 1: Table S5, Baseline characteristics). Similar to the discovery cohort, there were no significant associations between hypothermia and mortality in elderly patients (JAAMSR, *P* = 0.34; SPH, *P* = 0.81) (Fig. 2b, c). Test for homogeneity of the effect of BT < 36.0 °C on mortality using a meta-analytic technique revealed no significant differences across all three cohorts (*I*^2^ = 17% in non-elderly patients) (Fig. 3).

The effects of HR > 90 beats/min and RR > 30 breaths/min on mortality observed in the elderly patients of the discovery cohort were not replicated in validation cohort 1 (HR > 90 beats/min, *P* = 0.82; RR > 30 breaths/min, *P* = 0.96).

### Analysis including additional covariates

Repeating the Cox regression analysis including age, sex, chronic steroid use, APACHE II score, and significantly different baseline parameters between non-elderly and elderly patients (FORECAST suspected site of infection, body mass index, Charlson comorbidity index; JAAMSR suspected site of infection, body mass index, comorbidities [stroke, heart failure, chronic lung disease]; SPH comorbidities [chronic hepatic disease, chronic lung disease] (Table 1 and Additional file 1: Table S4 and Table S5)) yielded the same results (Additional file 2: Figure S1).

### Analysis using different age cutoffs

We performed the sensitivity analysis for the association of BT < 36.0 °C and mortality using different age cutoff values of 65, 70, and 80 years. The age cutoffs of 70 and 80 years revealed similar results to those observed in the primary analysis. However, the effect of hypothermia was eliminated using a cutoff of 65 years (Additional file 3: Figure S2).

### Analysis of fever, the interaction of age and BT, and 28-day in-hospital mortality

Because hypothermia had effects on mortality across all three cohorts, we tested for the effect of fever in all three cohorts in the secondary analysis. In non-elderly patients, a trend towards reduced hazard of death was observed when BT > 38.3 °C in the discovery cohort (*P* = 0.11, adjusted hazard ratio 0.71, 95% CI 0.47–1.08) (Fig. 1 and Additional file 4: Figure S3a). This potential effect was highly significant in both validation cohorts (JAAMSR, *P* = 0.0043, adjusted hazard ratio 0.51, 95% CI 0.32–0.81; SPH, *P* = 0.018, adjusted hazard ratio 0.76, 95% CI 0.61–0.95) (Additional file 4: Figure S3b-c). A meta-analytic test for heterogeneity revealed no significant difference in effect across cohorts (*I*^2^ = 14%) and a significant combined effect (*P* = 0.0006, adjusted hazard ratio 0.70, 95% CI 0.57–0.86) (Additional file 5: Figure S4). However, no significant effect of fever was observed in elderly patients in any cohort (FORECAST, *P* = 0.71; JAAMSR, *P* = 0.50; SPH, *P* = 0.72) (Additional file 4: Figure S3a-c).

The interaction of age (non-elderly or elderly) and hypothermia (BT < 36.0 °C or ≥ 36.0 °C) for mortality was statistically significant across the three cohorts (FORECAST, *P* = 0.0059; JAAMSR, *P* = 0.15; SPH, *P* = 0.012; three cohorts combined, *P* = 0.0001). The interaction term between the age and fever (BT ≤ 38.3 °C or > 38.3 °C) for mortality was also significant across the three cohorts (FORECAST, *P* = 0.23; JAAMSR, *P* = 0.13; SPH, *P* = 0.033; three cohorts combined, *P* = 0.005). These results indicate that the BT response (both low and high) has a significant impact on mortality in non-elderly patients but does not appear to have an impact on elderly patients.

Repeating analysis using 28-day mortality yielded similar, but partly stronger relationships between vital signs and mortality as those observed in the primary analysis (Additional file 6: Figure S5).

## Discussion

In the present study, non-elderly sepsis patients with hypothermia (BT < 36.0 °C) had significantly increased mortality, and those with fever (BT ≥ 38.3 °C) had decreased mortality. In contrast, hypothermia and fever were not associated with altered mortality in elderly patients. Interestingly, the other vital sign values were not consistently associated with differences in the outcome in patients of any age. Thus, BT was distinct among the vital signs for its association with the clinical outcomes in the non-elderly, but not elderly, patients with sepsis.

The key finding of our analysis is that the association of BT alterations on mortality was only observed in the younger septic population as opposed to elderly patients. The meta-analytic technique showed that there was low heterogeneity (*I*^2^ = 17%) in the association between hypothermia and mortality in non-elderly patients in the three cohorts (Fig. 3). In a secondary analysis, the opposite effect of fever was also only observed in non-elderly sepsis patients (Additional file 4: Figure S3 and Additional file 5: Figure S4). The changes in the association between the vital signs and mortality based on age have been investigated in several categories of critically ill patients. In blunt trauma, the presenting vital signs are less predictive of mortality in elderly patients as compared to the non-elderly [14]. Additionally, vital signs are less predictive of in-hospital cardiac arrest in elderly patients as compared to the non-elderly [15]. Since the significant associations of tachycardia and tachypnea on mortality in elderly patients were not replicated in the validation cohorts, we confirmed that BT alterations are uniquely predictive of mortality among the vital signs in non-elderly patients with sepsis but not in the elderly patients. Significant interactions with mortality between hypothermia or fever and age groups reinforced these findings.

In the current understanding of pathomechanism of sepsis-related BT changes, fever and hypothermia both have adaptive biological value for the host. In this context, fever is considered as an indicator of active (disease-fighting) strategy which may be beneficial in diseases of mild-to-moderate severity in a previously healthy host while hypothermia is a passive (disease-tolerating) strategy that is advantageous in severe forms of the disease, especially in the presence of comorbidities [29–31]. Nevertheless, BT is not the cause but rather the indicator of the severity, thus consequently of the outcome of the disease. In our previous study employing the FORECAST and JAAMSR cohorts, with no distinction between the non-elderly and elderly sepsis patients, hypothermia was associated with poor clinical outcomes [32, 33]. In another study of adult sepsis patients, hypothermia (< 36.0 °C) within 24 h from sepsis diagnosis was associated with a higher 28-day and 1-year mortality [34]. Oppositely, increased BT in sepsis/septic shock patients admitted to an ICU within 24 h of hospital arrival was associated with decreased mortality [35]. These reports were in line with our results in non-elderly patients.

Several factors could explain the difference in the relationships between the hypothermia and clinical outcome in elderly and non-elderly patients with sepsis. The physiology of fever generation and development of hypothermia is complicated; however, reduced production of pyrogenic cytokines such as IL-6 and TNF-a may be related to the lower incidence of fever seen in elderly septic patients [36, 37]. Comorbidities such as stroke, which was a higher prevalence in the elderly group in this study, may comprise neurological deficits that could impair thermoregulatory/inflammatory reflexes [38, 39]. Decreased heat production due to reduced muscle mass and increased heat radiation due to a reduced fat mass and blunted peripheral vasoconstriction capacity might be related to the lower BTs [40]. Observational studies have indeed reported that higher age populations have lower BTs and smaller diurnal BT variations [41, 42], which is consistent with our results. Lower BT and altered BT response in elderly septic patients as the result of age-related physical and functional deterioration may mitigate the hypothermic/febrile effects on clinical outcomes.

There were several limitations to the present study. First was the descriptive nature of the observational study. The primary findings were validated using two independent cohorts, which included multiple centers and patients of different ancestry; however, these do not prove a causal link. Second limitation of the study was the absence of data on variables that may have confounded BT measurement, including temperature measurement site and whether patients received antipyretics or targeted temperature management. Third was on the retrieval of vital signs. The vital signs corresponding to the APACHE II score were analyzed in the present study. However, according to the APACHE II scoring, a temperature as lower than 34 °C receives 1 point, whereas fever higher than 38.9 °C receives 3 points. Therefore, patients presenting both hypothermia and fever were more likely to be categorized in the fever group, which may have influenced the results. In addition, since the analyzed data were only in the first 24 h after the diagnosis of sepsis, it is unclear whether the duration of hypothermia or temperature changes over time affects the outcome. Another limitation could be the absence of established criteria for defining elderly patients. In this study, we applied the currently accepted standards in literature [20–22]. The analyses in the age cutoff of 70 and 80 years yielded the same conclusion, which could help strengthen the results of this study.

## Conclusions

We investigated the difference in the associations of the vital signs with mortality between non-elderly and elderly patients with sepsis. Among the vital signs, only BT showed significantly different effects on mortality between non-elderly and elderly patients. Hypothermia is associated with increased mortality and fever with decreased mortality in non-elderly septic patients. In contrast, hypothermia and fever did not impact mortality in elderly patients.

## Supplementary information

**Additional file 1: Table S1.** Cut-off values of the vital signs. **Table S2.** Correlation analysis between APACHE II score and vital signs. **Table S3.** Baseline characteristics and clinical outcomes between the subgroups of 90-day in-hospital survivors and non-survivors in nonelderly and elderly patients of derivation cohort (FORECAST cohort). **Table S4.** Baseline characteristics and clinical outcomes in validation cohort 1 (JAAMSR cohort). **Table S5.** Baseline characteristics and clinical outcomes in validation cohort 2 (SPH cohort).

**Additional file 2: Figure S1.** Associations between the vital signs and 90-day in-hospital mortality adjusted with additional covariates which associated with age related-changes. **a.** Discovery analysis (FORECAST cohort). **b.** Validation analyses (JAAMSR cohort and SPH cohort).

**Additional file 3: Figure S2.** Associations between the body temperature < 36.0 °C and 90-day in-hospital mortality using different age cut-offs. **a.** 65 years cut-off. **b.** 70 years cut-off. **c.** 80 years cut-off.

**Additional file 4: Figure S3.** Probability of mortality corresponding to the body temperature category of > 38.3 °C. **a.** Discovery cohort (FORECAST cohort). **b.** Validation cohort 1 (JAAMSR cohort). **c.** Validation cohort 2 (SPH cohort).

**Additional file 5: Figure S4.** Meta-analysis to test for the homogeneity between each studies for association of body temperature > 38.3 °C and 90-day in-hospital mortality.

**Additional file 6: Figure S5.** Associations between the vital signs and 28-day in-hospital mortality in discovery cohort (FORECAST cohort).

## Data Availability

The datasets analyzed during the current study are available with the corresponding author on reasonable request.

## Abbreviations

BP: Blood pressure
BT: Body temperature
FORECAST: Focused Outcomes Research in Emergency Care in Acute Respiratory Distress Syndrome, Sepsis, and Trauma
HR: Heart rate
JAAM: Japanese Association for Acute Medicine
JAAMSR: Japanese Association for Acute Medicine Sepsis Registry
MAP: Mean arterial pressure
RR: Respiratory rate
SBP: Systolic blood pressure
SPH: St. Paul’s Hospital
