# Supplementary material for: Significance of body temperature in elderly patients with sepsis
Source: Crit Care. 2020 Jun 30;24:387. doi: 10.1186/s13054-020-02976-6 (PMC7329464; doi:10.1186/s13054-020-02976-6)
Supplement: Supplementary file 1 — Additional file 1: Table S1. Cut-off values of the vital signs. Table S2. Correlation analysis between APACHE II score and vital signs. Table S3. Baseline characteristics and clinical outcomes between the subgroups of 90-day in-hospital survivors and non-survivors in nonelderly and elderly patients of derivation cohort (FORECAST cohort). Table S4. Baseline characteristics and clinical outcomes in validation cohort 1 (JAAMSR cohort). Table S5. Baseline characteristics and clinical outcomes in validation cohort 2 (SPH cohort). [file 13054_2020_2976_MOESM1_ESM.docx]

**Significance of body temperature in elderly patients with sepsis**

Takashi Shimazui, Taka-aki Nakada, Keith R. Walley, Taku Oshima, Toshikazu Abe, Hiroshi Ogura, Atsushi Shiraishi, Shigeki Kushimoto, Daizoh Saitoh, Seitaro Fujishima, Toshihiko Mayumi, Yasukazu Shiino, Takehiko Tarui, Toru Hifumi, Yasuhiro Otomo, Kohji Okamoto, Yutaka Umemura, Joji Kotani, Yuichiro Sakamoto, Junichi Sasaki, Shin-ichiro Shiraishi, Kiyotsugu Takuma, Ryosuke Tsuruta, Akiyoshi Hagiwara, Kazuma Yamakawa, Tomohiko Masuno, Naoshi Takeyama, Norio Yamashita, Hiroto Ikeda, Masashi Ueyama, Satoshi Fujimi, Satoshi Gando, on behalf of the JAAM FORECAST Study Group

***Online data Supplement***

**Table S1.** Cut-off values of the vital signs

|  | Cut-off values | Criteria |
| --- | --- | --- |
| Body temperature | <36.0 °C | Sepsis-2 |
|  | >38.3 °C | Sepsis-2 |
| Heart rate | >90 beats/min | Sepsis-2 |
| Systolic blood pressure | <90 mmHg | Sepsis-2 |
|  | ≤100 mmHg | Sepsis-3 |
| Mean arterial pressure | <65 mmHg | Sepsis-3 |
|  | <70 mmHg | Sepsis-2 |
| Respiratory rate | ≥22 breaths/min | Sepsis-3 |
|  | >30 breaths/min. | Sepsis-2 |

Cut-off values of the vital signs were selected according to the sepsis-2 and sepsis-3 criteria [1, 2].

**Table S2.** Correlation analysis between APACHE II score and vital signs

|  | Correlation coefficient |
| --- | --- |
| Body temperature | -0.058 |
| Heart rate | 0.22 |
| Systolic blood pressure | -0.36 |
| Mean arterial pressure | -0.37 |
| Respiratory rate | 0.20 |

Since APACHE II score may correlate to vital signs, Pearson correlation coefficients between APACHE II score and vital signs were analyzed. The correlation coefficients, ranged from -0.37 and 0.22, indicated low correlation.

**Table S3.** Baseline characteristics and clinical outcomes between the subgroups of 90-day in-hospital survivors and non-survivors in nonelderly and elderly patients of derivation cohort (FORECAST cohort)

|  | Nonelderly | |  | Elderly | |  |
| --- | --- | --- | --- | --- | --- | --- |
|  | Survivor  (n = 505) | Non-survivors  (n = 123) | *P* value | Survivor  (n = 390) | Non-survivors  (n = 130) | *P* value |
| Characteristics |  |  |  |  |  |  |
| Age, yr | 65 (53–69) | 67 (61–71) | 0.0038 | 82 (78-86) | 83 (79-87) | 0.48 |
| Male sex, n (%) | 325 (64.4) | 80 (65.0) | 0.89 | 215 (55.1) | 73 (56.2) | 0.84 |
| Suspected site of infection, n (%) |  |  |  |  |  |  |
| Lung | 139 (27.5) | 54 (43.9) | 0.0004 | 116 (29.7) | 48 (36.9) | 0.13 |
| Intra-abdominal | 125 (24.8) | 25 (20.3) | 0.30 | 115 (29.5) | 30 (23.1) | 0.16 |
| Urinary tract | 95 (18.8) | 7 (5.7) | 0.0004 | 98 (25.1) | 18 (13.8) | 0.0075 |
| Soft tissue | 67 (13.3) | 12 (9.8) | 0.29 | 23 (5.9) | 12 (9.2) | 0.19 |
| Others^a^ | 79 (15.6) | 25 (20.3) | 0.21 | 38 (9.7) | 22 (16.9) | 0.026 |
| Septic Shock, n (%) | 293 (58.0) | 94 (76.4) | 0.0002 | 236 (60.5) | 95 (73.1) | 0.010 |
| Body mass index | 22.5 (19.5–25.3) | 22.1 (18.6–25.1) | 0.58 | 20.8 (18.6-23.7) | 20.7 (18.8-24.6) | 0.46 |
| Chronic steroid use, n (%) | 60 (11.9) | 26 (21.1) | 0.0074 | 37 (9.5) | 18 (13.8) | 0.16 |
| Comorbidity, n (%) |  |  |  |  |  |  |
| Diabetes mellitus | 121 (24.0) | 34 (27.6) | 0.40 | 74 (19.0) | 33 (25.4) | 0.12 |
| Stroke | 45 (8.9) | 10 (8.1) | 0.78 | 58 (14.9) | 20 (15.4) | 0.89 |
| Malignancy | 71 (14.1) | 30 (24.4) | 0.0052 | 59 (15.1) | 18 (13.8) | 0.72 |
| Heart failure | 36 (7.1) | 13 (10.6) | 0.20 | 51 (13.1) | 23 (17.7) | 0.19 |
| Chronic kidney disease | 28 (5.5) | 11 (8.9) | 0.16 | 21 (5.4) | 20 (15.4) | 0.0002 |
| Liver disease | 25 (5.0) | 18 (14.6) | 0.0001 | 15 (3.8) | 10 (7.7) | 0.076 |
| Chronic lung disease | 27 (5.3) | 12 (9.8) | 0.069 | 26 (6.7) | 15 (11.5) | 0.074 |
| Charlson comorbidity index | 1 (0-2) | 2 (1-3) | <0.0001 | 1 (0-2) | 2 (1-3) | 0.0050 |
| SOFA score | 8 (5–11) | 11 (8–14) | <0.0001 | 8 (5-11) | 11 (8-13) | <0.0001 |
| APACHE II score | 20 (15–27) | 29 (24–36) | <0.0001 | 22 (16-28) | 29 (22-35) | <0.0001 |
| Vital signs on day 1^b^ |  |  |  |  |  |  |
| Body temperature, °C | 38.0 (36.9–39.0) | 37.6 (36.2–39.0) | 0.040 | 37.6 (36.7-38.5) | 37.6 (36.6-38.5) | 0.50 |
| Heart rate, beats/min | 113 (96–131) | 120 (96–140) | 0.054 | 106 (90-121) | 113 (100-130) | 0.0009 |
| Mean arterial pressure, mmHg | 65 (55–81) | 57 (47–69) | <0.0001 | 64 (53-78) | 59 (50-71) | 0.0023 |
| Systolic blood pressure, mmHg | 88 (73–113) | 79 (65–94) | <0.0001 | 90 (74-114) | 82 (66-100) | 0.0019 |
| Respiratory rate, breath/min | 25 (20–31) | 26 (21–34) | 0.33 | 25 (20-30) | 29 (22-36) | 0.0005 |

Median (inter quartile range)

SOFA, sequential organ failure assessment

APACHE, acute physiology and chronic health evaluation

^a^: Including central nervous system, catheter related, osteoarticular, endocardium, wound, implant device related, and undifferentiated infection.

^b^: Most abnormal value corresponding to the APACHE II score.

*P* values were calculated using Pearson’s chi-square test and Mann-Whitney U test.

**Table S4.** Baseline characteristics and clinical outcomes in validation cohort 1 (JAAMSR cohort)

|  | Nonelderly (<75 yr)  (n = 348) | Elderly (≥75 yr)  (n = 276) | *P* value |
| --- | --- | --- | --- |
| Characteristics |  |  |  |
| Age, yr | 62 (50–69) | 82 (79–86) | <0.0001 |
| Male, n (%) | 239 (68.7) | 152 (55.1) | 0.0005 |
| Suspected site of infection, n (%) |  |  |  |
| Lung | 137 (39.4) | 124 (44.9) | 0.16 |
| Intra-abdominal | 70 (20.1) | 63 (22.8) | 0.41 |
| Urinary tract | 30 (8.6) | 48 (17.4) | 0.0010 |
| Soft Tissue | 51 (14.7) | 27 (9.8) | 0.068 |
| Others^a^ | 60 (17.2) | 14 (5.1) | <0.0001 |
| Septic Shock, n (%) | 164 (47.1) | 118 (42.8) | 0.28 |
| Body mass index | 22.0 (19.1–25.0) | 21.1 (18.2–23.8) | 0.015 |
| Chronic steroid use, n (%) | 44 (12.6) | 21 (7.6) | 0.041 |
| Comorbidity, n (%) |  |  |  |
| Diabetes mellitus | 77 (22.1) | 45 (16.3) | 0.069 |
| Stroke | 23 (6.6) | 68 (24.6) | <0.0001 |
| Malignancy | 44 (12.6) | 32 (11.6) | 0.69 |
| Heart failure | 20 (5.7) | 31 (11.2) | 0.013 |
| Chronic kidney disease | 26 (7.5) | 17 (6.2) | 0.52 |
| Liver disease | 22 (6.3) | 11 (4.0) | 0.20 |
| Chronic lung disease | 9 (2.6) | 16 (5.8) | 0.042 |
| SOFA score | 9 (5–11) | 8 (6–11) | 0.84 |
| APACHE II score | 21 (16–28) | 24 (19–29) | 0.0003 |
| Vital signs on day 1^b^ |  |  |  |
| Body temperature, °C | 38.2 (37.0–39.0) | 37.4 (35.8–38.3) | <0.0001 |
| Heart rate, beats/min | 120 (105–140) | 114 (96–130) | 0.0003 |
| Mean arterial pressure, mmHg | 59 (48–73) | 60 (50–73) | 0.41 |
| Systolic blood pressure, mmHg | 82 (66–102) | 82 (70–104) | 0.46 |
| Respiratory rate, breath/min | 28 (23–34) | 27 (22–32) | 0.16 |
| Outcome |  |  |  |
| 28-day in-hospital mortality, n (%) | 62 (17.8) | 78 (28.3) | 0.0019 |
| 90-day in-hospital mortality, n (%) | 85 (24.4) | 91 (33.0) | 0.018 |

Median (inter quartile range)

SOFA, sequential organ failure assessment

APACHE, acute physiology and chronic health evaluation

^a^: Including central nervous system, catheter related, osteoarticular, endocardium, wound, implant device related, and undifferentiated infection.

^b^: Most abnormal value corresponding to the APACHE II score.

*P* values were calculated using Pearson’s chi-square test and Mann-Whitney U test.

**Table S5.** Baseline characteristics and clinical outcomes in validation cohort 2 (SPH cohort)

|  | Nonelderly (<75 yr)  (n = 833) | Elderly (≥75 yr)  (n = 171) | *P* value |
| --- | --- | --- | --- |
| Characteristics |  |  |  |
| Age, yr | 56 (44–66) | 79 (77–82) | <0.0001 |
| Male, n (%) | 536 (64.3) | 117 (68.4) | 0.31 |
| Septic Shock, n (%) | 567 (68.1) | 129 (75.4) | 0.057 |
| Chronic steroid use, n (%) | 66 (7.9) | 5 (2.9) | 0.020 |
| Comorbidity, n (%) |  |  |  |
| Chronic heart disease | 53 (6.4) | 14 (8.2) | 0.38 |
| Chronic kidney disease | 60 (7.2) | 7 (4.1) | 0.14 |
| Chronic hepatic disease | 93 (11.2) | 4 (2.3) | 0.0004 |
| Chronic lung disease | 145 (17.4) | 41 (24.0) | 0.044 |
| APACHE II score | 23 (17–29) | 26 (19–33) | 0.0021 |
| Vital signs on day 1^a^ |  |  |  |
| Body temperature, °C | 38.0 (37.0–38.6) | 37.5 (36.2–38.5) | 0.0010 |
| Heart rate, beats/min | 115 (95–130) | 105 (95–120) | 0.0029 |
| Mean arterial pressure, mmHg | 58 (52–67) | 55 (50–62) | 0.0003 |
| Outcome |  |  |  |
| 28-day in-hospital mortality, n (%) | 299 (35.9) | 102 (59.6) | <0.0001 |
| 90-day in-hospital mortality, n (%) | 353 (42.4) | 115 (67.3) | <0.0001 |

Median (inter quartile range)

APACHE: acute physiology and chronic health evaluation

^a^: Most abnormal value corresponding to the APACHE II score.

*P* values were calculated using Pearson’s chi-square test and Mann-Whitney U test.

**References**

1. Levy MM, Fink MP, Marshall JC, Abraham E, Angus D, Cook D, et al. 2001 SCCM/ESICM/ACCP/ATS/SIS International Sepsis Definitions Conference. Intensive Care Med. 2003;29:530-8.

2. Singer M, Deutschman CS, Seymour CW, Shankar-Hari M, Annane D, Bauer M, et al. The Third International Consensus Definitions for Sepsis and Septic Shock (Sepsis-3). JAMA. 2016;315:801-10.
