# Supplementary material for: Significance of body temperature in elderly patients with sepsis
Source: Crit Care. 2020 Jun 30;24:387. doi: 10.1186/s13054-020-02976-6 (PMC7329464; doi:10.1186/s13054-020-02976-6)
Supplement: Supplementary file 2 — Additional file 2: Figure S1. Associations between the vital signs and 90-day in-hospital mortality adjusted with additional covariates which associated with age related-changes. a. Discovery analysis (FORECAST cohort). b. Validation analyses (JAAMSR cohort and SPH cohort). [file 13054_2020_2976_MOESM2_ESM.pdf]

Figure S1. Associations between the vital signs and 90-day in-hospital mortality adjusted with additional covariates which associated with age related-changes

a. Discovery analysis (FORECAST cohort)

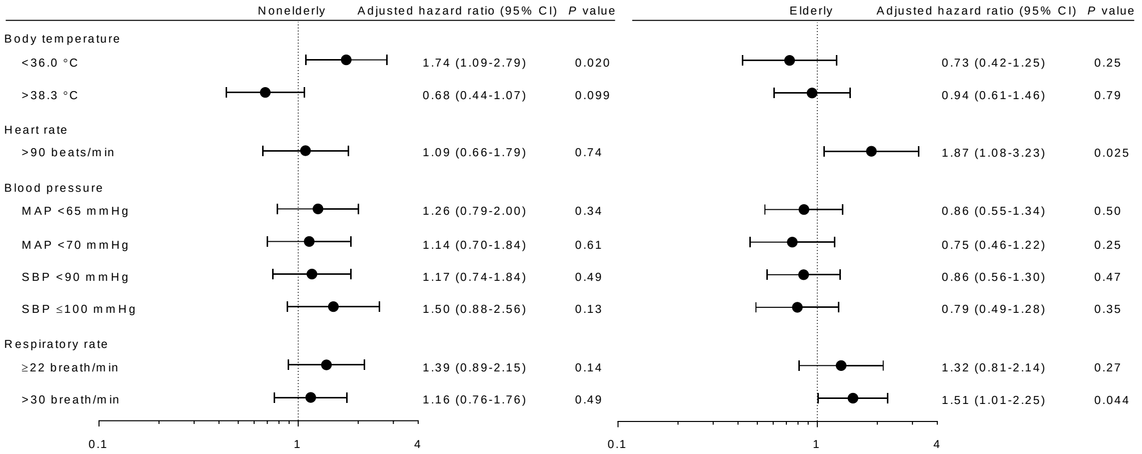

b. Validation analyses (JAAMSR cohort and SPH cohort)

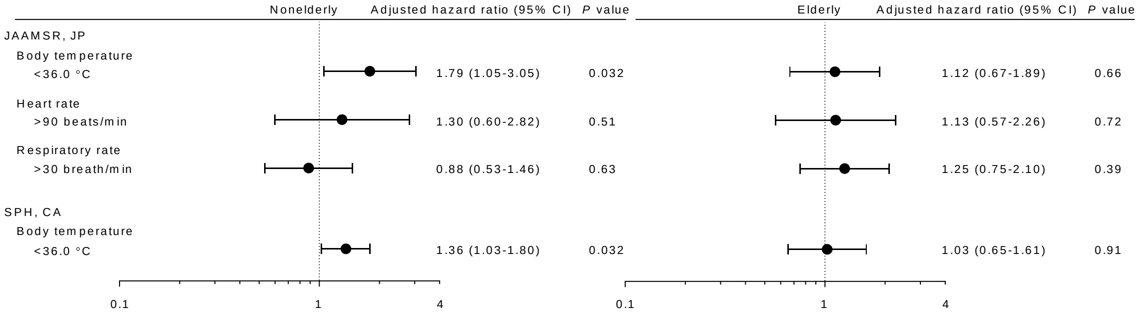

Nonelderly patients with BT <36.0 °C showed a significant increase for hazard of death over 90-day period in all three cohorts.

JP, Japan, CA, Canada, CI, confidence interval.

The adjusted hazard ratio was calculated using age, sex, chronic steroid use, acute physiology, and chronic health evaluation (APACHE) II score, and significantly different baseline parameters between nonelderly and elderly patients (FORECAST suspected site of infection, body mass index, Charlson comorbidity index; JAAMSR suspected site of infection, body mass index, comorbidities [stroke, heart failure, chronic lung disease]; SPH comorbidities [chronic hepatic disease, chronic lung disease]).
