# Supplementary material for: Significance of body temperature in elderly patients with sepsis
Source: Crit Care. 2020 Jun 30;24:387. doi: 10.1186/s13054-020-02976-6 (PMC7329464; doi:10.1186/s13054-020-02976-6)
Supplement: Supplementary file 3 — Additional file 3: Figure S2. Associations between the body temperature < 36.0 °C and 90-day in-hospital mortality using different age cut-offs. a. 65 years cut-off. b. 70 years cut-off. c. 80 years cut-off. [file 13054_2020_2976_MOESM3_ESM.pdf]

Figure S2. Associations between the body temperature <36.0 °C and 90-day in-hospital mortality using different age cut-offs.

a. 65 years cut-off

Study or Subgroup	log[Hazard Ratio]	SE	Weight	Hazard Ratio IV, Random, 95% CI
**1.1.1 Age <65 yr, BT<36.0 °C**				
FORECAST, JP	1.34645	0.409285	27.4%	3.84 [1.72, 8.57]
JAAMSR, JP	0.084858	0.36351	30.2%	1.09 [0.53, 2.22]
SPH, CA	0.282817	0.179777	42.3%	1.33 [0.93, 1.89]
**Subtotal (95% CI)**			**100.0%**	**1.67 [0.89, 3.16]**
Heterogeneity: Tau² = 0.22; Chi² = 6.52, df = 2 (P = 0.04); I² = 69%				
Test for overall effect: Z = 1.59 (P = 0.11)				
**1.1.2 Age ≧65 yr, BT<36.0 °C**				
FORECAST, JP	-0.071782	0.197173	30.7%	0.93 [0.63, 1.37]
JAAMSR, JP	0.503032	0.184837	32.6%	1.65 [1.15, 2.38]
SPH, CA	0.279291	0.160685	36.7%	1.32 [0.96, 1.81]
**Subtotal (95% CI)**			**100.0%**	**1.28 [0.94, 1.74]**
Heterogeneity: Tau² = 0.04; Chi² = 4.57, df = 2 (P = 0.10); I² = 56%				
Test for overall effect: Z = 1.55 (P = 0.12)				

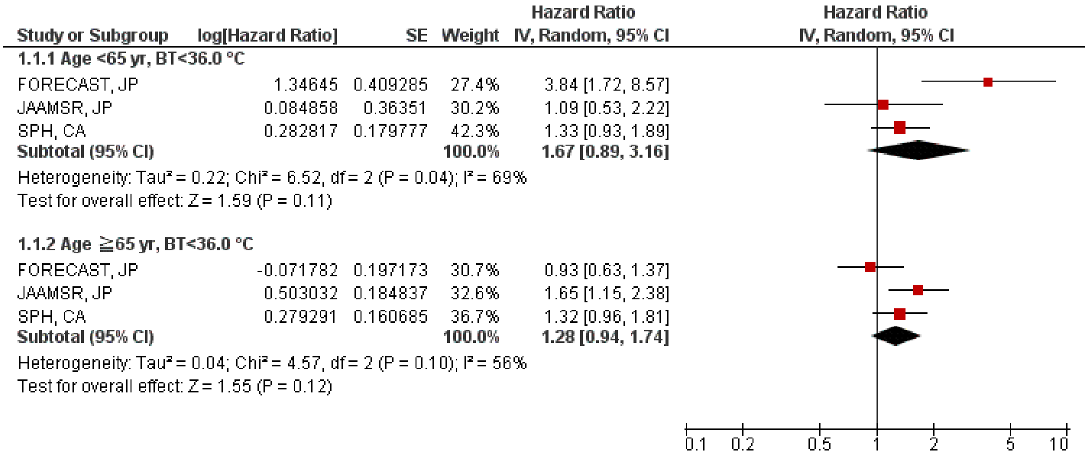

b. 70 years cut-off

Study or Subgroup	log[Hazard Ratio]	SE	Weight	Hazard Ratio IV, Random, 95% CI
**1.1.1 Age <70 yr, BT<36.0 °C**				
FORECAST, JP	1.068481	0.295817	29.5%	2.91 [1.63, 5.20]
JAAMSR, JP	0.787702	0.277686	30.8%	2.20 [1.28, 3.79]
SPH, CA	0.243395	0.161537	39.6%	1.28 [0.93, 1.75]
**Subtotal (95% CI)**			**100.0%**	**1.92 [1.14, 3.25]**
Heterogeneity: Tau² = 0.15; Chi² = 7.29, df = 2 (P = 0.03); I² = 73%				
Test for overall effect: Z = 2.46 (P = 0.01)				
**1.1.2 Age ≧70 yr, BT<36.0 °C**				
FORECAST, JP	-0.286607	0.223578	30.2%	0.75 [0.48, 1.16]
JAAMSR, JP	0.24586	0.203654	33.0%	1.28 [0.86, 1.91]
SPH, CA	0.275202	0.178581	36.9%	1.32 [0.93, 1.87]
**Subtotal (95% CI)**			**100.0%**	**1.10 [0.78, 1.55]**
Heterogeneity: Tau² = 0.05; Chi² = 4.44, df = 2 (P = 0.11); I² = 55%				
Test for overall effect: Z = 0.56 (P = 0.58)				

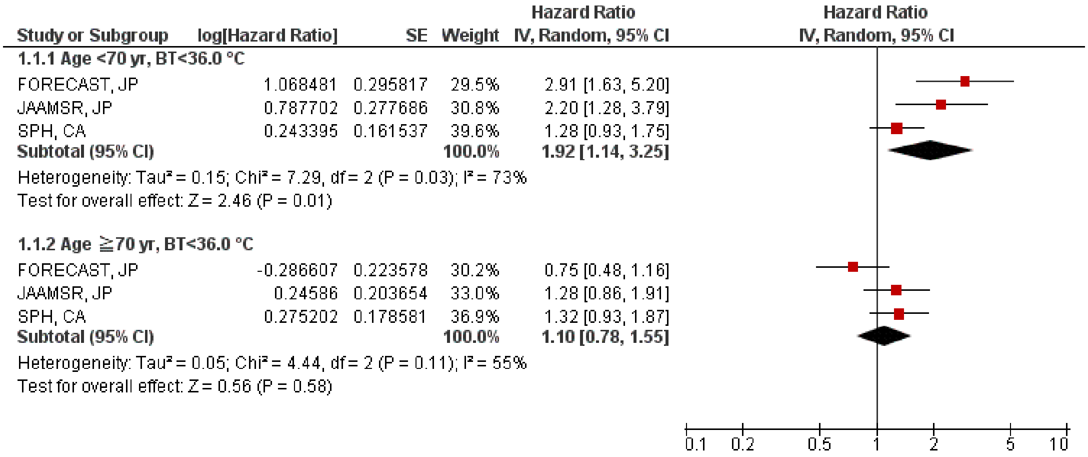

c. 80 years cut-off

Study or Subgroup	log[Hazard Ratio]	SE	Weight	Hazard Ratio IV, Random, 95% CI
**1.1.1 Age <80 yr, BT<36.0 °C**				
FORECAST, JP	0.435948	0.206006	22.8%	1.55 [1.03, 2.32]
JAAMSR, JP	0.421579	0.216087	20.8%	1.52 [1.00, 2.33]
SPH, CA	0.248018	0.131071	56.4%	1.28 [0.99, 1.66]
**Subtotal (95% CI)**			**100.0%**	**1.39 [1.14, 1.68]**
Heterogeneity: Tau² = 0.00; Chi² = 0.83, df = 2 (P = 0.66); I² = 0%				
Test for overall effect: Z = 3.32 (P = 0.0009)				
**1.1.2 Age ≧80 yr, BT<36.0 °C**				
FORECAST, JP	-0.721298	0.365	29.7%	0.49 [0.24, 0.99]
JAAMSR, JP	0.449075	0.249869	36.9%	1.57 [0.96, 2.56]
SPH, CA	0.040078	0.305888	33.4%	1.04 [0.57, 1.90]
**Subtotal (95% CI)**			**100.0%**	**0.97 [0.51, 1.84]**
Heterogeneity: Tau² = 0.23; Chi² = 7.01, df = 2 (P = 0.03); I² = 71%				
Test for overall effect: Z = 0.11 (P = 0.91)				

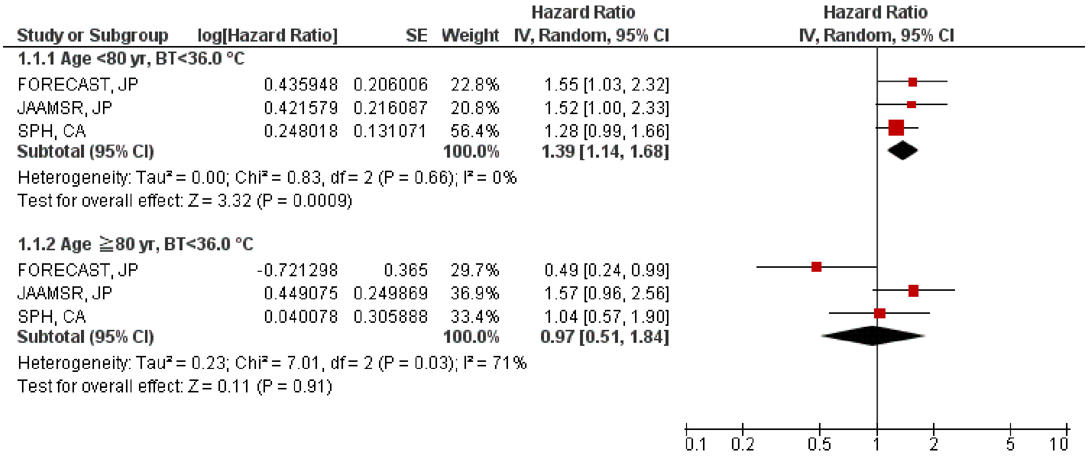

The age cut-off of 70 and 80 years revealed similar results to those observed in the primary analysis. However, the effect of hypothermia was eliminated using a cut-off of 65 years.
JP, Japan; CA, Canada; CI, confidence interval.
The hazard ratio from each cohort was calculated with adjusting the potentially confounding factors such as the age, sex, chronic steroid use, and acute physiology and chronic health evaluation (APACHE) II score.
