# Supplementary material for: Significance of body temperature in elderly patients with sepsis
Source: Crit Care. 2020 Jun 30;24:387. doi: 10.1186/s13054-020-02976-6 (PMC7329464; doi:10.1186/s13054-020-02976-6)
Supplement: Supplementary file 4 — Additional file 4: Figure S3. Probability of mortality corresponding to the body temperature category of > 38.3 °C. a. Discovery cohort (FORECAST cohort). b. Validation cohort 1 (JAAMSR cohort). c. Validation cohort 2 (SPH cohort). [file 13054_2020_2976_MOESM4_ESM.pdf]

Figure S3. Probability of mortality corresponding to the body temperature category of >38.3 °C

a. Discovery cohort (FORECAST cohort)

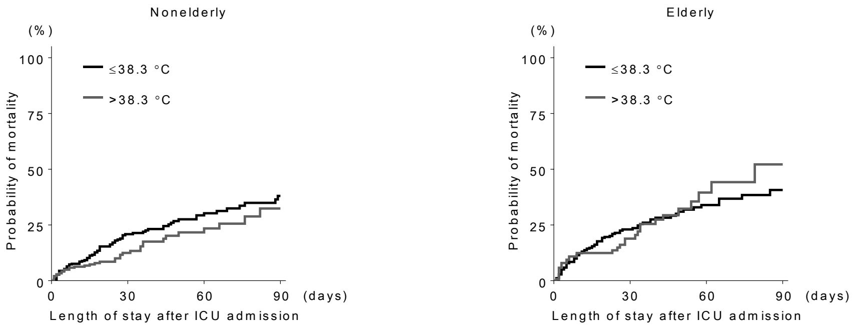

Number at risk

	Nonelderly 0	30	60	90	Elderly 0	30	60	90
≤38.3 °C	363	157	74	39	358	138	57	25
>38.3 °C	243	103	44	15	141	54	13	4

b. Validation cohort 1 (JAAMSR cohort)

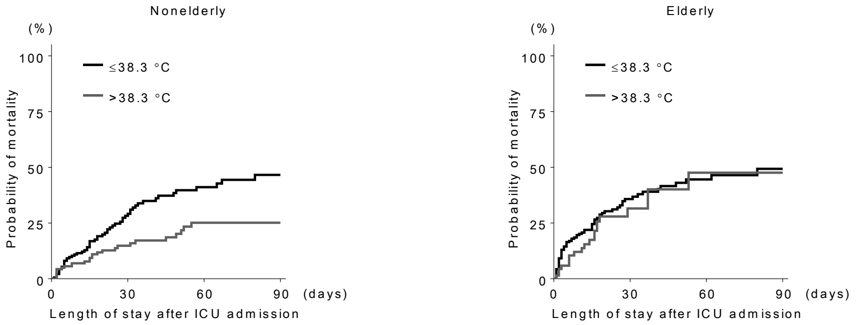

Number at risk

	Nonelderly 0	30	60	90	Elderly 0	30	60	90
≤38.3 °C	187	79	42	23	208	62	29	16
>38.3 °C	161	76	35	19	68	19	5	2

c. Validation cohort 2 (SPH cohort)

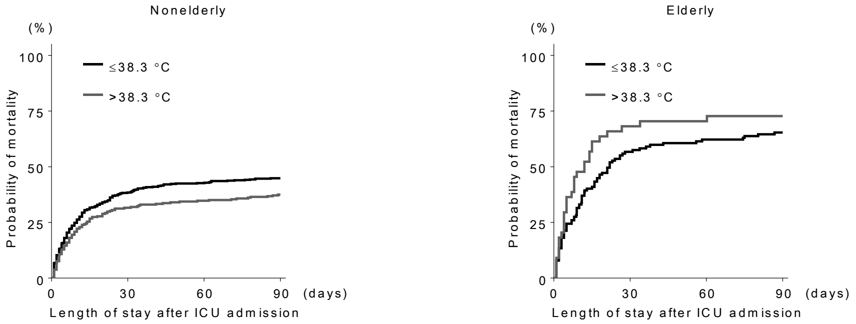

Number at risk

	Nonelderly 0	30	60	90	Elderly 0	30	60	90
≤38.3 °C	544	335	312	300	127	55	48	44
>38.3 °C	288	197	188	180	44	14	13	12

Nonelderly patients with fever had lower mortality in all three cohorts.
