# Supplementary material for: Significance of body temperature in elderly patients with sepsis
Source: Crit Care. 2020 Jun 30;24:387. doi: 10.1186/s13054-020-02976-6 (PMC7329464; doi:10.1186/s13054-020-02976-6)
Supplement: Supplementary file 5 — Additional file 5: Figure S4. Meta-analysis to test for the homogeneity between each studies for association of body temperature > 38.3 °C and 90-day in-hospital mortality. [file 13054_2020_2976_MOESM5_ESM.pdf]

Figure S4. Meta-analysis to test for the homogeneity between each studies for association of body temperature >38.3 °C and 90-day in-hospital mortality.

Study or Subgroup	log[Hazard Ratio]	SE	Weight	Hazard Ratio IV, Random, 95% CI
**1.1.1 Nonelderly, BT>38.3 °C**				
FORECAST, JP	-0.33651	0.212173	22.1%	0.71 [0.47, 1.08]
JAAMSR, JP	-0.672075	0.235139	18.4%	0.51 [0.32, 0.81]
SPH, CA	-0.273765	0.115887	59.5%	0.76 [0.61, 0.95]
**Subtotal (95% CI)**			**100.0%**	**0.70 [0.57, 0.86]**
Heterogeneity: Tau² = 0.01; Chi² = 2.31, df = 2 (P = 0.31); I² = 14%				
Test for overall effect: Z = 3.42 (P = 0.0006)				
**1.1.2 Eldelry, BT>38.3 °C**				
FORECAST, JP	-0.080464	0.214886	37.2%	0.92 [0.61, 1.41]
JAAMSR, JP	-0.16993	0.254843	26.4%	0.84 [0.51, 1.39]
SPH, CA	0.078125	0.217297	36.4%	1.08 [0.71, 1.66]
**Subtotal (95% CI)**			**100.0%**	**0.95 [0.74, 1.23]**
Heterogeneity: Tau² = 0.00; Chi² = 0.59, df = 2 (P = 0.75); I² = 0%				
Test for overall effect: Z = 0.35 (P = 0.72)				

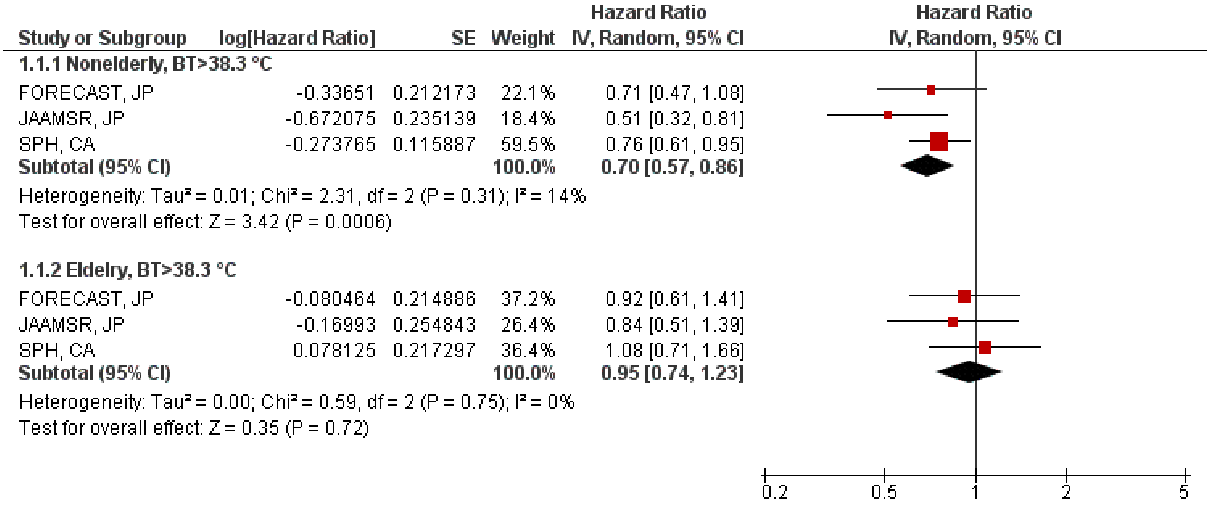

In nonelderly patients, fever had did not have significant effect on mortality in the discovery cohort, but presented a significant combined effect (I^2=14%, *P*=0.0006, adjusted hazard ratio 0.70, 95% CI 0.57-0.86). Fever had no significant effect on mortality in elderly patients in the individual cohorts or their combination.

JP, Japan; CA, Canada; CI, confidence interval.

The hazard ratio from each cohort was calculated with adjusting the potentially confounding factors such as the age, sex, chronic steroid use, and acute physiology and chronic health evaluation (APACHE) II score.
