# Supplementary material for: Significance of body temperature in elderly patients with sepsis
Source: Crit Care. 2020 Jun 30;24:387. doi: 10.1186/s13054-020-02976-6 (PMC7329464; doi:10.1186/s13054-020-02976-6)
Supplement: Supplementary file 6 — Additional file 6: Figure S5. Associations between the vital signs and 28-day in-hospital mortality in discovery cohort (FORECAST cohort). [file 13054_2020_2976_MOESM6_ESM.pdf]

Figure S5. Associations between the vital signs and 28-day in-hospital mortality in discovery cohort (FORECAST cohort)

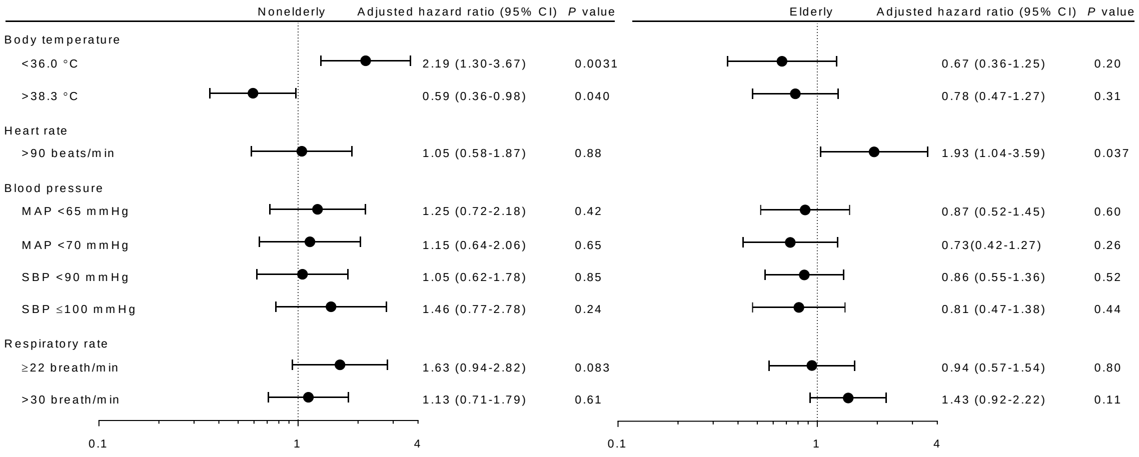

Nonelderly patients with BT <36.0 °C showed a significant increase, and with BT >38.3 °C showed a significant decrease for hazard of death over 28-day period. Hypothermia and fever were not associated with mortality in elderly patients. Heart rate >90 beats/min had a significant association with increased mortality in elderly patients.

CI, confidence interval.

The adjusted hazard ratio was calculated using potentially confounding factors such as age, sex, chronic steroid use, and acute physiology and chronic health evaluation (APACHE) II score.
